# Two convergent pathways of DNA knotting in replicating DNA molecules as revealed by θ-curve analysis

**DOI:** 10.1093/nar/gky559

**Published:** 2018-06-30

**Authors:** Danielle O’Donnol, Andrzej Stasiak, Dorothy Buck

**Affiliations:** 1 Department of Mathematics, Indiana University Bloomington, 831 E. Third Street, Bloomington, IN 47405, USA; 2 Center for Integrative Genomics, University of Lausanne,1015 Lausanne, Switzerland; 3 Swiss Institute of Bioinformatics, 1015 Lausanne, Switzerland; 4 Centre for Mathematical Biology, and Department of Mathematical Sciences, University of Bath, North Rd, Bath BA2 7AY, England

## Abstract

During DNA replication in living cells some DNA knots are inadvertently produced by DNA topoisomerases facilitating progression of replication forks. The types of DNA knots formed are conditioned by the 3D organization of replicating DNA molecules. Therefore, by characterizing formed DNA knots it is possible to infer the 3D arrangement of replicating DNA molecules. This topological inference method is highly developed for knotted DNA circles. However, partially replicated DNA molecules have the form of θ-curves. In this article, we use mathematical formalism of θ-curves to characterize the full possibilities of how knotting can occur during replication of DNA molecules *in vivo*. To do this, we reanalyze earlier experimental studies of knotted, partially replicated DNA molecules and the previously proposed pathway of their formation. We propose a general model of knotting in replication intermediates, and demonstrate that there is an additional, equally important, parallel knotting pathway that also explains how DNA topoisomerases can produce experimentally observed knotted θ-curves. Interestingly, both pathways require intertwining of freshly replicated sister duplexes (*precatenanes*).

## INTRODUCTION

The shapes of DNA knots formed *in vivo* or *in vitro* are tale-tellers both about the mechanism of the DNA topoisomerases (1–3) or site-specific DNA recombinases that produced these DNA knots (4–10) and also about specific arrangements of DNA molecules at the moment they were knotted (11–14). When DNA knots formed *in vivo* are analysed after their isolation from cells, they still contain the same topological information. The knot types remains unchanged even after knotted DNA molecules are spread, covered with RecA protein (15) and adsorbed to supporting films for electron microscopy characterization (4) or alternately loaded on agarose gels to recognise these knots by their characteristic electrophoretic migration (5,16,17).

Electron microscopy studies showed that depending on the EM preparation method utilised, the partially replicated DNA plasmids can have either a form with freshly replicated sister duplexes winding around each other forming precatenanes or a form without precatenanes (18). Sogo *et al.* then identified that knotting occurs in partially replicated DNA molecules. They characterized the types of knots formed *in vivo* and concluded that the formed knots demonstrate that *in vivo* freshly replicated portions of partially replicated DNA molecules wind around each other forming precatenanes (14). Sogo *et al.* presented a model for knotting arising from intersegmental passages within the same sister duplex. In this article, we propose a general model of knotting arising during replication that involves both the original model and passages occurring between two different sister duplexes that form precatenanes in partially replicated DNA molecules. Interestingly, the topological consequences of intra- and inter-sister passages are the same.

Since from a mathematical point of view, partially replicated DNA molecules form θ-curves, our topological analysis of knotted replication bubbles is based on newly characterized topologies of θ-curves (arXiv:1710.05237v2, Buck D. and O’Donnol, D. with Appendix by Baker, K. *Unknotting numbers for prime θ-curves up to 7 crossings*).

## MATERIALS AND METHODS

In this article we model replication intermediates (RIs) with embedded θ-graphs equivalent up to ambient isotopy. The formation of knotting is modeled by a crossing change. In this section we will give definitions and outline the pertinent mathematics.

### Equivalence and diagrams

The θ-graph is the graph with two vertices and three edges between them. We study θ-curves, which are embedded θ-graphs up to *ambient isotopy*. Roughly speaking, this means that a graph can be continuously distorted and we still consider it the same graph. However, it cannot be cut or passed through itself. A knot is an embedded circle, also equivalent up to ambient isotopy. So we can think of knots as subgraphs of our θ-curves. Each pair of edges of the θ-graph form a cycle, these are called constituent knots.

**Figure 1.**
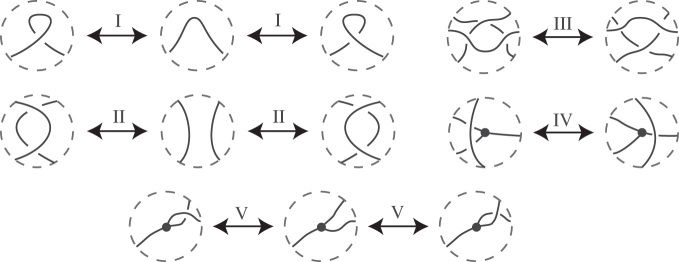
The Reidemeister moves that can be applied to diagrams to θ-curves. Moves I, II and III are the same as for knots. In move IV an arc passes a vertex either over or behind (not shown). In move V the edges incident to a vertex switch places.

We work with diagrams of θ-curves, which are projections to the plane where there are only double points away from vertices, with over and under crossings indicated. There are many different diagrams for a given θ-curve. If two graph diagrams are of the same θ-curve then there exists a finite sequence of Reidemeister moves (and planar isotopy) from one diagram to the other (19). Reidemeister moves are changes of the original diagram that can change the number of visible crossings or the place where crossings occur but do not change the underlying topology. These moves can be of different types: i.e. local twisting or untwisting, or moving a portion of the diagram over another portion. In 1927 Kurt Reidemeister demonstrated that all topology-preserving deformations of diagrams of knots can be decomposed into a sequence of just three elementary types of moves (20) that today are called Reidemeister moves I, II and III, respectively (see Figure 1). For θ-curves, there are two additional types of topology-preserving moves called Reidemeister moves IV and V. In Figure 1, we show the five types of Reidemeister moves. For each type we show how the diagram changes, whereas the rest of the diagram (which is not pictured) does not change. For general information on the Reidemeister moves for knots see (21).

### Prime knots and θ-curves

Both knots and θ-curves can be prime and composite. For the purposes of this article we will only be working with prime knots and prime θ-curves. For more information about prime and connect sum θ-curves see (22).

**Figure 2.**
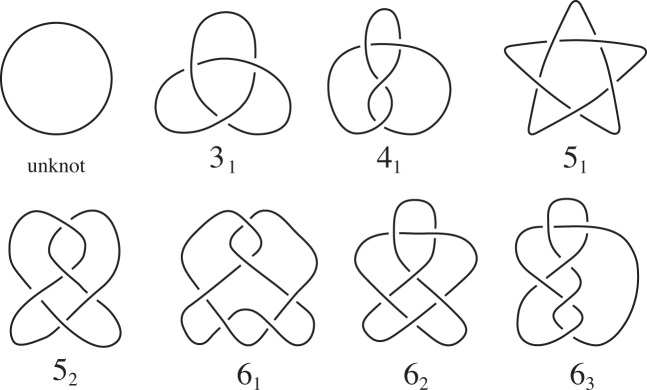
The prime knots up to six crossings. Knots $\overline{3_1}, 4_1, \overline{5_2}$ and $\overline{6_2}$ were observed by Sogo *et al.* (14) to form within replication bubble of partially replicated DNA molecules.

Hoste–Thistlethwaite have compiled tables of all of the prime knots up to 16 crossings (https://github.com/dimpase/knotscap). For knots we will use the names in Rolfsen’s tables (21). For convenience, we have a table of all the prime knots up to six crossings in Figure 2. The prime θ-curves with up to seven crossings were originally compiled by Litherland. They were later published and verified by Moriuchi, (23). We will use the names given in this table. (See Supplementary Figure S1.) Though it should be clear from context, to further distinguish θ-curves from knots, the θ-curve names appear in **bold** with a θ at the start.

### Chirality of knots and θ-curves

Given a knot *K*, the mirror image of *K*, denoted $\overline{K}$, is the knot whose diagram is obtained by changing every crossing in a diagram of *K*. A knot *K* is achiral if it is equivalent to its mirror image $\overline{K}$. Otherwise it is chiral. Among the knots with six crossings or less, the unknot, 4_1_ and 6_3_ are achiral. As with knots, given a θ-curve θ, the mirror image of θ, denoted $\overline{\theta }$, is the graph whose diagram is obtained by changing every crossing in a diagram of θ. A θ-curve θ is achiral if it is equivalent to its mirror image $\overline{\theta }$.

### Unknotting number

The enzyme DNA Topoisomerase IV (Topo IV), which is responsible for the knotting in the RIs in *Escherichia coli*, is known to act by single DNA–DNA passages (24). This is modeled with a crossing change: in a knot a segment is passed through another segment, or in a graph an edge is passed through itself or another edge. In a diagram a crossing change can be made at a crossing of the diagram. In this case, the segments at that crossing are passed through each other resulting in moving the under-segment to the over-segment and *vice versa*.

The trivial knot (also called the unknot) is a circle. Similarly, the trivial θ-curve is the planar θ-curve (up to equivalence). We will denote the trivial θ-curve as $\theta \mathbf {0_1}$. The unknotting number of a θ-curve θ, *u*(θ), is the minimal number of crossing changes i.e. of intersegmental passages, needed to convert that θ-curve into $\theta \mathbf {0_1}$.

All θ-curves with unknotting number 1 can be created from the trivial θ-curve by a single crossing change resulting from single action of Topo IV. However, θ-curves with higher unknotting numbers require more crossing changes and thus more rounds of Topo IV mediated passages to be created from $\theta \mathbf {0_1}$.

**Figure 3.**
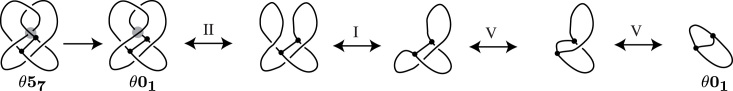
The θ-curve $\theta \mathbf {5_7}$ with unknotting crossing change. The arrow indicates the highlighted crossing change, and the double arrows are the indicated Reidemeister moves.

Buck and O’Donnol (with an appendix by Baker) have determined *u*(*g*) for all of the θ-curves in the Litherland-Moriuchi Table (arXiv:1710.05237v2). In Supplementary Figure S1, we indicate a set of crossing changes that will unknot each θ-curve. The unknotting crossing changes are highlighted in gray. For example, Figure 3 shows how $\theta \mathbf {5_7}$ can be converted to $\theta \mathbf {0_1}$ after the indicated crossing change.

## RESULTS

In this section we present a general model for how Topo IV-driven knotting occurs after one or more passages during DNA replication. Our model includes the pathway introduced in (14), and is based on electron microscopy characterization of DNA knots formed *in vivo* in partially replicated DNA molecules. Sogo *et al.* utilized electrophoretic separation followed by electron microscopy to characterize knots formed within so-called replication bubbles. This involved cutting the unreplicated portion of partially replicated DNA molecules with restriction enzymes. Therefore there was no information gathered about possible knotting events resulting from passages between replicated and unreplicated portions of partially replicated DNA molecules. Sogo *et al.*, used gel electrophoresis to separate knotted replication bubbles according to their expected number of crossings. Electron microscopy studies of DNA contained in DNA bands expected to contain knots with 3, 4, 5 and 6 crossings revealed that the first band contained only right-handed trefoil knots $\overline{3_1}$ and the second achiral 4_1_ knots. Knots characterized in the next two bands were right-handed $\overline{5_2}$ and right-handed $\overline{6_2}$ knot, respectively.

Subsequently, Lopez *et al.* analyzed intact RIs via electrophoretic separation followed by atomic force microscopy (AFM) (24). There was no indication of intersegmental passages between replicated and unreplicated portions of partially replicated DNA molecules, based on their analysis. The AFM imaging revealed structures of the form $\theta \mathbf {0_1}$ and $\theta \mathbf {3_1}$.

### A model for Topo IV-driven knotting in replication intermediates (RI) due to a single passage

We will model a RI with a θ-curve. Vertices are at the replication forks, and edges represent segments of duplex DNA. The enzyme Topo IV, which is responsible for the knotting in the RIs in *E. coli* (24), acts via an intersegmental passage. This means that the knotting can be modeled by one or more crossing changes on the corresponding θ-curve. First we will consider the model for a single passage.

The previous proposed pathway of knotting in RIs was introduced in (14), and further discussed in (12,24).

**Figure 4.**
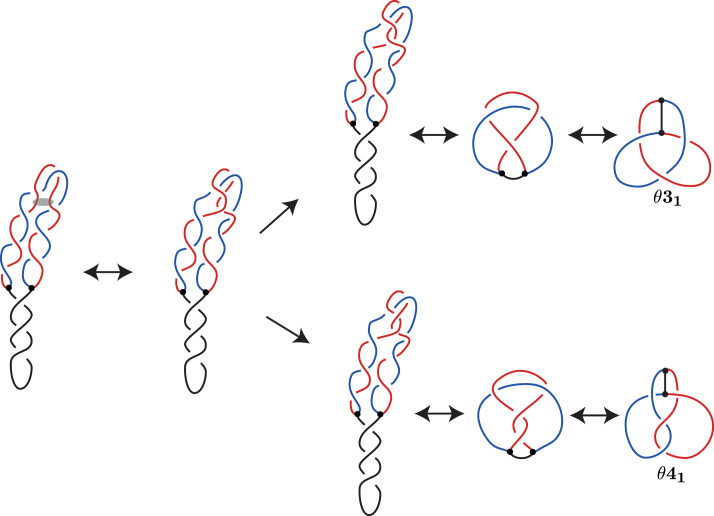
Previous proposed pathway of knotting in RIs. The DNA–DNA passage occurs between portions of the same sister duplex. The semi-transparent oval in the first diagram indicates portions of freshly replicated duplexes that will serve as substrate of Topo IV-mediated passages in this case. Notice that both indicated portions belong to the same freshly replicated sister duplex (drawn in red).

In Figure 4, we show in detail the proposed pathway of knotting of (14). We start with an idealized, very regular configuration of partially replicated, supercoiled DNA molecules. Due to negative supercoiling, introduced by DNA gyrase, the unreplicated portion winds plectonemically around itself. In contrast, freshly replicated sister duplexes (indicated in blue and red) wind around each other in a left-handed direction, as expected for negatively supercoiled partially replicated DNA molecules (18). For the sake of clarity, we present the freshly replicated, precatenated portions as forming a planar bend. However, in reality these portions may form higher order plectonemes as demonstrated by Stone *et al.* (25). Such plectonemes make it even more likely that Topo IV will perform passages between portions of freshly replicated duplexes. Next, we introduce some thermal fluctuations that in this case cause overlaps between segments belonging to the same freshly replicated sister duplex. There are two ways the passages could occur in the region of overlap.

A DNA–DNA passage within the same sister duplex could occur at one of two crossings, following the upper or lower pathway shown in Figure 4. In each pathway, the double arrows indicate Reidemeister moves or planar isotopies between equivalent diagrams, first moving to a minimal crossing diagram and then to the diagram shown in the Litherland–Moriuchi Table (Supplementary Figure S1).

### DNA–DNA passage can occur between the two sister duplexes

**Figure 5.**
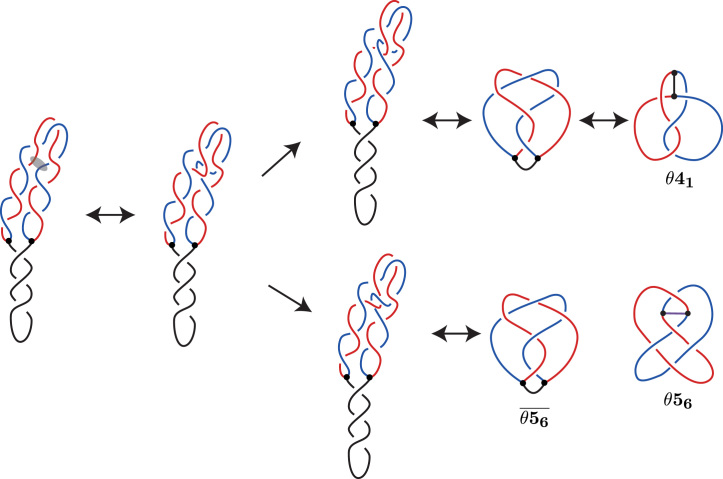
The new (second) pathway for knotting in RIs. The strand passage occurs between the two sister duplexes. Similarly to Figure 4, the semi-transparent oval in the first diagram indicates portions of freshly replicated duplexes that will serve as substrate of Topo IV-mediated passages in the analyzed here case. Notice that both indicated portions belong to two different freshly replicated sister duplexes (drawn in red and blue).

We propose a second pathway where Topo IV can mediate DNA–DNA passages between portions of the two sister duplexes, rather than between portions of the same sister duplex. See Figure 5.

Similarly to Figure 4, we start with an idealized, regular configuration of a negatively supercoiled partially replicated DNA molecule, in which freshly replicated sister duplexes wind around each other in a left-handed way. Similarly to the situation presented in Figure 4, we introduce fluctuations that cause overlaps between segments belonging to different sister duplexes (indicated in blue and red). The overlapping regions with two crossings can serve then as a substrate for Topo IV that performs DNA–DNA passages. The resulting topological outcomes of passages occurring at these two crossings differ from each other and are presented in Figure 5 in the upper or lower route.

In each route, the double arrows indicate Reidemiester moves and planar isotopies between equivalent diagrams, first moving to a minimal crossing diagram and then to the diagram (or mirror of that) shown in the Litherland–Moriuchi Table (Supplementary Figure S1). Notice that the knot $\overline{5_2}$ is obtained from $\overline{\theta \mathbf {5_6}}$ by removing the arc which in our case corresponds to unreplicated portion of RI. The knot $\overline{5_2}$ was observed experimentally after cutting off the unreplicated portion of partially replicated DNA molecules (14).

### Both pathways produce the same set of knotted RIs

**Figure 6.**
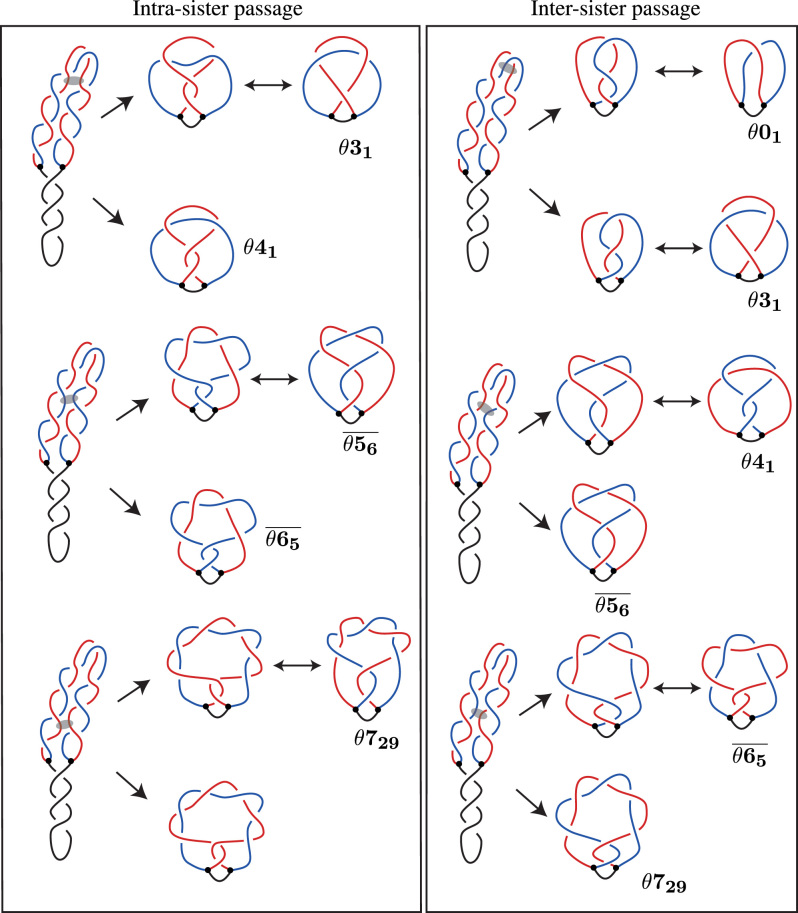
Our general model of knotting in RIs, with a comparison between the two pathways. The left column shows the possible resulting RIs from the previous pathway of knotting up to eight crossings. The right column shows the possible resulting RIs from our new pathway of knotting up to seven crossings. Like in Figures 4 and 5 the semi-transparent ovals indicate portions of freshly replicated duplexes that will serve as substrate of Topo IV mediated DNA–DNA passages in respective analyzed cases. (The complex knotted θ-curve that is shown in the lower part of the left panel is not labeled because the Litherland–Moriuchi Table is limited to θ-curves with up to seven crossings.)

Figure 6 shows the knotted RI that result for each pathway; the left column shows the result of a single passage between portions of the same sister duplex, and the right column shows the result of a single passage between portions of the two sister duplexes. Notice that depending on the number of windings between two sister duplexes occurring in the DNA portions separating the regions where the action of Topo IV occurred, the resulting topology can be different. In particular, more windings separating the regions of Topo IV action results in more complex knotted θ-curves. Regardless of whether the overlapping DNA segments belong to the same or different sister duplexes, the set of knotted θ-curves is the same.

### How the knot $\overline{6_2}$ could arise in the freshly replicated portion from a knot via one crossing change

Sogo *et al.* analyzed electron microscopy images of DNA knots formed in freshly replicated portions of the RI. In their experiment they digested the RI, cutting the non-replicated portion. We want to now focus on the freshly replicated portion which is one of the constituents of our RI; for ease of discussion we will call this constituent knot the FR knot. The FR knot is the single non-trivially knotted constituent containing both sister duplexes (14). In (14), they observed one FR knot, $\overline{6_2}$, that was not predicted by their model.

**Figure 7.**
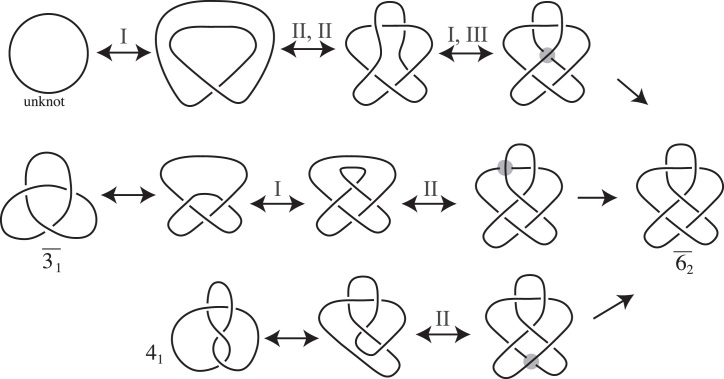
Three ways $\overline{6_2}$ can be formed via a single crossing change. This shows how one crossing change on the unknot, $\overline{3_1}$ or 4_1_ can result in the knot $\overline{6_2}$. The double arrows indicate moves that do not change the knot type; they are the Reidemeister moves I, II and III and planar isotopies. The single arrow indicates when a crossing change is made at the highlighted crossing.

To understand how $\overline{6_2}$ could arise as a constituent of a knotted RI, we show how $\overline{6_2}$ could arise from simpler knots via a single crossing change. We demonstrate that the knot $\overline{6_2}$ can be obtained from the unknot, $\overline{3_1}$ and 4_1_ via one crossing change (see Figure 7). The double arrows indicate moves that do not change the knot type; they are Reidemeister moves of types I, II or III, sets of Reidemeister moves, and planar isotopy. The single arrow indicates a crossing change that happens at the indicated crossing.

### Models for the formation of a $\overline{6_2}$ FR knot from an RI

Building on our understanding from the previous section, we now consider how $\overline{6_2}$ may arise as a FR knot.

**Figure 8.**
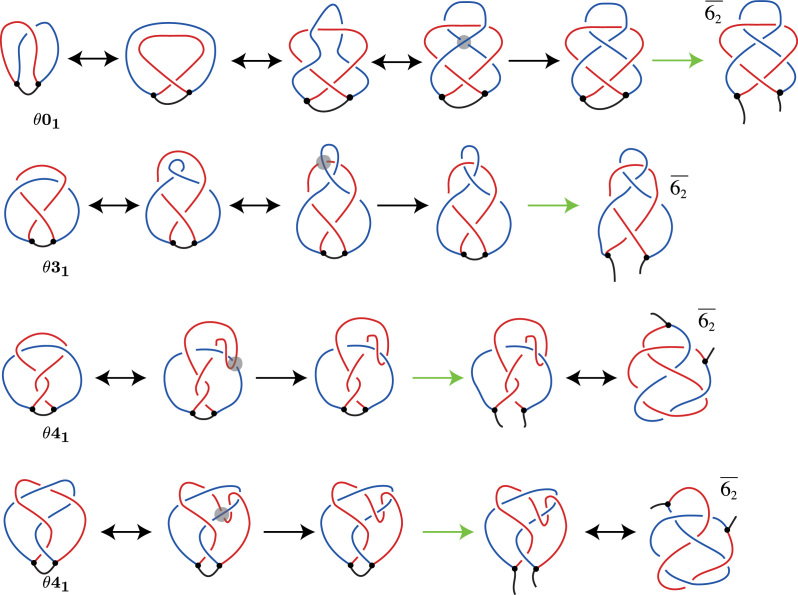
Four possible pathways of formation of $\overline{6_2}$ knots within replication bubbles of RIs. The top row shows one passage leading from unknotted replication bubble to the replication bubble forming $\overline{6_2}$ knot. The second row demonstrates a DNA–DNA passage on a $\theta \mathbf {3_1}$ RI that results in a $\overline{6_2}$ FR knot, as proposed by (14). The bottom two rows demonstrate how a DNA–DNA passage on a $\theta \mathbf {4_1}$ RI can result in a $\overline{6_2}$ FR knot. The third row starts with a $\theta \mathbf {4_1}$ RI formed via intra-sister DNA–DNA passage, where the fourth starts with a $\theta \mathbf {4_1}$ RI formed via inter-sister DNA–DNA passage. The double arrows indicate when the RI or digested RI is moved. The black arrows indicate a strand passage and the green arrows indicate the parent strand being cut.

In Figure 8, the first row shows how a $\overline{6_2}$ FR knot can arise by one DNA–DNA passage from an unknotted RI. We can see that such single-passage pathway is possible. However, it does not start from a RI in which freshly replicated portions are strongly precatenated in a left handed way, as is shown in Figures 6 and 7. This pathway rather requires torsionally relaxed structure of RI. In (14), Sogo *et al.* suggested that the $\overline{6_2}$ FR knot could be a result of a second segment passage on a $\theta \mathbf {3_1}$ RI, where one sister duplex forms a loop and then a DNA–DNA passage occurs between the loop and the other sister duplex. This is shown in the second row of Figure 8. The topology of $\theta \mathbf {4_1}$ is different depending on whether intra-sister or inter-sister DNA–DNA passage occurred in its formation; a pathway is shown for each topology. The third row starts with a $\theta \mathbf {4_1}$ RI formed via intra-sister DNA–DNA passage, where the fourth starts with a $\theta \mathbf {4_1}$ RI formed via inter-sister DNA–DNA passage.

## DISCUSSION

**Figure 9.**
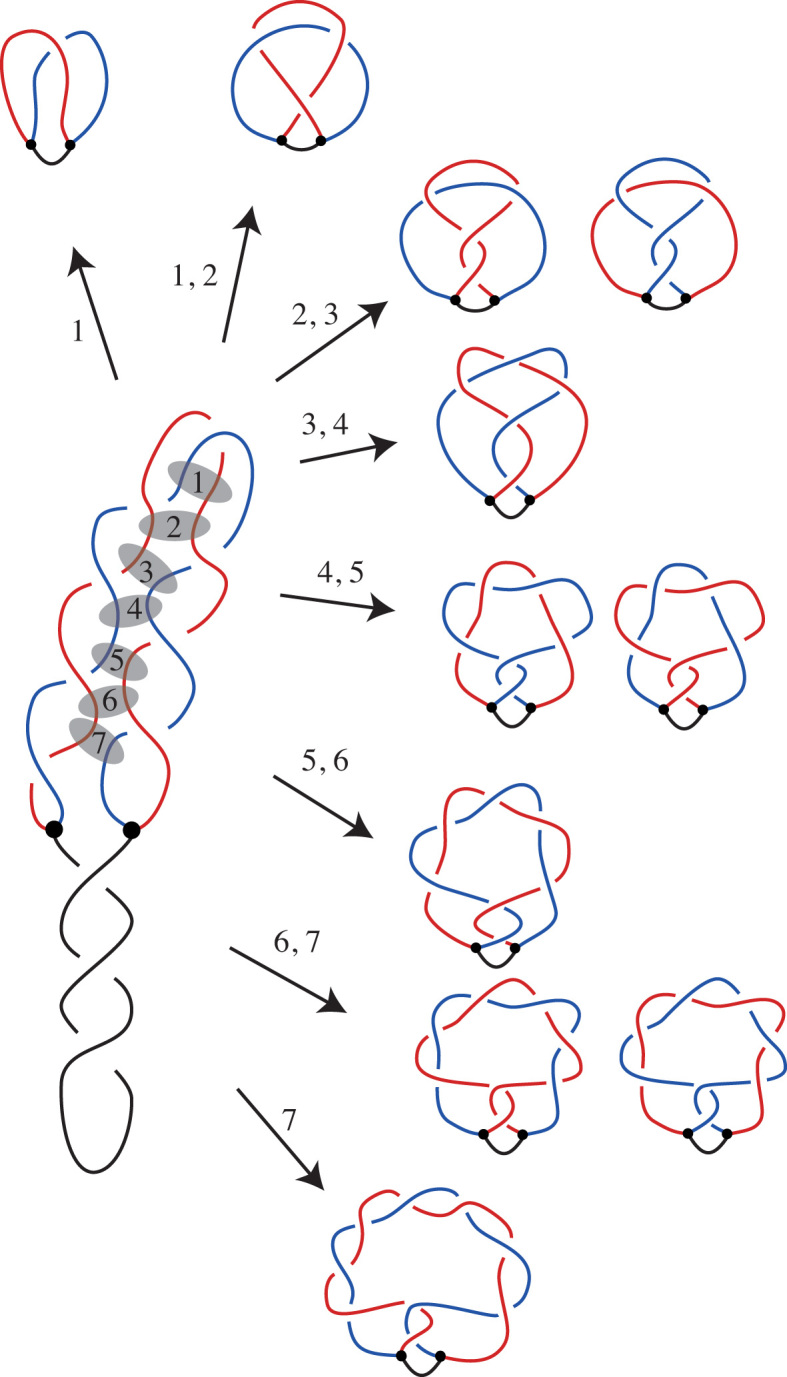
General model of DNA knotting in replicating DNA molecules. Depending on where Topo IV acts within partially replicated DNA molecules one obtains different knotted θ-curves. However, the set of of knotted θ-curves is the same for reactions involving segments of the same freshly replicated sister duplex (indicated with semi-transparent ovals 2, 4 and 6) or involving segments of different sister duplexes (indicated with ovals 1, 3, 5 and 7). (We do not provide notations for more complex knotted θ-curves that are shown in the lower part of the figure as the Litherland–Moriuchi Table is limited to θ-curves with up to seven crossings.) The unknotted θ-curve is shown here as resulting from passages involving segments of two different sister duplexes (oval 1). However, unknotted θ-curves can also result from passages between closely spaced segments located within the same sister duplex. When there are no crossings trapped between the regions of passages the knots do not form. The most complex θ-curve presented in the figure resulted from an inter-sister passage (oval 7). However, the same type of knotted θ-curves would be obtained by passages occurring between segments belonging to the same sister but located still closer to the replication forks of the RI drawn here.

Using mathematical formalism of knotted θ-curves, we have fully characterized the mechanism of formation of DNA knots in partially replicated DNA molecules. Our analysis revealed that characteristic types of DNA knots made *in vivo* in partially replicated DNA molecules can be formed by two pathways. The first pathway is the same as that proposed earlier by Sogo *et al.* (14). In this pathway, crossing changes may happen between segments of the same edge of the θ-curve, which in a biological setting correspond to Topo IV-mediated passage of segments from the same freshly replicated sister duplex within a partially replicated circular DNA molecule. However, there is also a different but equally likely second pathway that can produce the same set of knot types as experimentally observed in partially replicated DNA molecules. In this pathway, crossing changes may happen between segments of different edges of the θ-curve, which in a biological setting correspond to Topo IV-mediated passage of segments from two different freshly replicated sister duplexes within a partially replicated circular DNA molecules. These two pathways can be grouped into a more general model that explains how twist knot θ-curves can be formed by one crossing change from an unknotted, negatively supercoiled θ-curve whose sister duplexes wind around each other in a left-handed direction (see Figure 9).

Our model proposes that the winding between the two sister duplexes causes this portion of the molecule to exhibit higher order coiling. This higher order coiling looks locally like a supercoiled DNA molecule with a superhelical apex. (For simplicity, in our figures we show this portion as merely bent in half.) Topo IV acting on such a structure can mediate passages between segments of two superhelical regions running side-by-side (see Figure 9). In the situation presented in Figure 9, Topo IV-mediated intersegmental passages trap precatenane crossings within the intervening portion of the molecule. As in the stalled RIs studied by Sogo *et al.*, these precatenane crossings have positive sign, so this sign is maintained in the crossings of resulting knotted θ-curve. The Topo IV-mediated passage by itself can introduce two positive or two negative crossings into the θ-curve.

Additionally, we characterized the formation of the $\overline{6_2}$ knot identified in the freshly replicated DNA in (14) (see Figure 8). In the first two rows, the DNA must form a loop before the DNA–DNA passage. In the third row, the DNA must make a U-turn before the DNA–DNA passage. In the fourth row, the DNA moves to overlap with the sister duplex that it is next to before the DNA–DNA passage.

The fact that the new second knotting pathway resulting from inter-sister passages gives the same topological outcomes as the first knotting pathway (considered previously) involving only intra-sister passages strengthens the evidence that freshly replicated sister duplexes are precatenated. Topo IV action occurs locally and therefore the enzyme is not able to ‘know’ whether the contacting DNA segments belong to the same or to two freshly replicated duplexes, as to distinguish them the enzyme would need to track along the entire distance separating the interacting DNA sites. Sogo *et al.* considered only passages occurring within the same sister duplex and explained formation of observed knots by assuming that partially replicated DNA molecules form precatenanes. However, Sogo *et al.* did not consider whether equally likely inter-sister passages are expected to produce the same set of knots starting from the same structure of partially replicated DNA molecules. *A priori* the set of knots produced by intra and inter-sister passages does not need to be the same. If the inter-sister passages resulted in structures that had not been observed this would indicated that the previous pathway also does not occur. We show here that this is not the case and that the structure of partially replicated DNA molecules involves topological symmetry of precatenation.

Although our study focuses only on the structure of RIs when the forks are stopped, it also reveals the mechanical properties of RIs that includes the formation of precatenanes formed under torsional stress. Our analysis was done based on the knots isolated from cells in which replication fork progression was stopped, and as the consequence the negative supercoiling was reintroduced into DNA by DNA gyrase. There were no comparable systematic studies of knots formed during unperturbed replication as the prerequisite for characterizing knots formed in RIs requires stopping all replication at the same sequence (14). However, it is likely that actively replicated DNA molecules accumulate positive supercoiling arising from active strand separation (26). If the magnitude of positive supercoiling in actively replicating DNA molecules is similar to the magnitude of negative supercoiling observed in molecules with stopped replication forks, then the expected arrangement of actively replicating partially replicated DNA molecules would have unreplicated portions forming left-handed plectonemes and freshly replicated sister duplexes forming right-handed precatenanes. In this case, the θ-curve structure of the RI would be expected to be the mirror image of those presented here.

In this article we elucidate all the possibilities of how knotting can occur during replication of DNA molecules *in vivo*. Our general model describes how a specific family of twist-like knotted θ-curves can form when Topo IV acts on supercoiled, partially replicated DNA molecules. This model shows similarity to Wasserman *et al.* proposal of how a specific family of twist-like knots can form when DNA topoisomerases act on supercoiled circular DNA molecules (27).

## Supplementary Material

Supplementary Data
