# Supplementary material for: Two convergent pathways of DNA knotting in replicating DNA molecules as revealed by θ-curve analysis
Source: Nucleic Acids Res. 2018 Jun 30;46(17):9181–8. doi: 10.1093/nar/gky559 (PMC6158496; doi:10.1093/nar/gky559)
Supplement: Supplementary Data [file gky559_supplemental_figures.pdf]

θ	Constituent Knots	u	θ	Constituent Knots	u	θ	Constituent Knots	u
$\theta\mathbf{3_1}$	2x0_1 3_1	1	$\theta\mathbf{7_6}$	2x0_1 3_1	1	$\theta\mathbf{7_{36}}$	0_1 5_1 7_3	2
$\theta\mathbf{4_1}$	2x0_1 4_1	1	$\theta\mathbf{7_7}$	2x0_1 3_1	1	$\theta\mathbf{7_{37}}$	0_1 5_2 7_3	2
$\theta\mathbf{5_1}$	3x0_1	1	$\theta\mathbf{7_8}$	0_1 2x3_1	2	$\theta\mathbf{7_{38}}$	2x0_1 7_4	2
$\theta\mathbf{5_2}$	2x0_1 3_1	1	$\theta\mathbf{7_9}$	0_1 2x3_1	1	$\theta\mathbf{7_{39}}$	2x0_1 7_4	2
$\theta\mathbf{5_3}$	2x0_1 5_1	2	$\theta\mathbf{7_{10}}$	0_1 2x3_1	2	$\theta\mathbf{7_{40}}$	0_1 3_1 7_4	2
$\theta\mathbf{5_4}$	0_1 3_1 5_1	2	$\theta\mathbf{7_{11}}$	2x0_1 5_2	1	$\theta\mathbf{7_{41}}$	0_1 3_1 7_4	2
$\theta\mathbf{5_5}$	2x0_1 5_2	1	$\theta\mathbf{7_{12}}$	2x0_1 4_1	1	$\theta\mathbf{7_{42}}$	0_1 5_2 7_4	2
$\theta\mathbf{5_6}$	2x0_1 5_2	1	$\theta\mathbf{7_{13}}$	2x0_1 4_1	1	$\theta\mathbf{7_{43}}$	2x0_1 7_5	2
$\theta\mathbf{5_7}$	0_1 3_1 5_2	1	$\theta\mathbf{7_{14}}$	0_1 2x4_1	1	$\theta\mathbf{7_{44}}$	2x0_1 7_5	2
$\theta\mathbf{6_1}$	3x0_1	1	$\theta\mathbf{7_{15}}$	2x0_1 5_1	2	$\theta\mathbf{7_{45}}$	0_1 3_1 7_5	2
$\theta\mathbf{6_2}$	2x0_1 3_1	1	$\theta\mathbf{7_{16}}$	2x0_1 5_1	2	$\theta\mathbf{7_{46}}$	0_1 3_1 7_5	2
$\theta\mathbf{6_3}$	0_1 3_1 4_1	1	$\theta\mathbf{7_{17}}$	2x0_1 5_1	2	$\theta\mathbf{7_{47}}$	0_1 3_1 7_5	2
$\theta\mathbf{6_4}$	0_1 3_1 4_1	1	$\theta\mathbf{7_{18}}$	0_1 5_1 5_2	2	$\theta\mathbf{7_{48}}$	0_1 5_1 7_5	2
$\theta\mathbf{6_5}$	2x0_1 6_1	1	$\theta\mathbf{7_{19}}$	2x0_1 5_2	1	$\theta\mathbf{7_{49}}$	0_1 5_2 7_5	2
$\theta\mathbf{6_6}$	2x0_1 6_1	1	$\theta\mathbf{7_{20}}$	2x0_1 5_2	1	$\theta\mathbf{7_{50}}$	2x0_1 7_6	1
$\theta\mathbf{6_7}$	2x0_1 6_1	1	$\theta\mathbf{7_{21}}$	2x0_1 5_2	1	$\theta\mathbf{7_{51}}$	2x0_1 7_6	1
$\theta\mathbf{6_8}$	0_1 4_1 6_1	1	$\theta\mathbf{7_{22}}$	0_1 3_1 5_2	2	$\theta\mathbf{7_{52}}$	2x0_1 7_6	1
$\theta\mathbf{6_9}$	2x0_1 6_2	1	$\theta\mathbf{7_{23}}$	0_1 4_1 5_2	2	$\theta\mathbf{7_{53}}$	2x0_1 7_6	1
$\theta\mathbf{6_{10}}$	2x0_1 6_2	1	$\theta\mathbf{7_{24}}$	0_1 4_1 5_2	2	$\theta\mathbf{7_{54}}$	2x0_1 7_6	1
$\theta\mathbf{6_{11}}$	2x0_1 6_2	1	$\theta\mathbf{7_{25}}$	2x0_1 7_1	3	$\theta\mathbf{7_{55}}$	0_1 3_1 7_6	2
$\theta\mathbf{6_{12}}$	0_1 3_1 6_2	2	$\theta\mathbf{7_{26}}$	0_1 3_1 7_1	3	$\theta\mathbf{7_{56}}$	0_1 3_1 7_6	1
$\theta\mathbf{6_{13}}$	0_1 4_1 6_2	1	$\theta\mathbf{7_{27}}$	0_1 5_1 7_1	3	$\theta\mathbf{7_{57}}$	0_1 4_1 7_6	2
$\theta\mathbf{6_{14}}$	2x0_1 6_3	1	$\theta\mathbf{7_{28}}$	2x0_1 7_2	1	$\theta\mathbf{7_{58}}$	0_1 5_2 7_6	2
$\theta\mathbf{6_{15}}$	2x0_1 6_3	1	$\theta\mathbf{7_{29}}$	2x0_1 7_2	1	$\theta\mathbf{7_{59}}$	2x0_1 7_7	1
$\theta\mathbf{6_{16}}$	0_1 3_1 6_3	1	$\theta\mathbf{7_{30}}$	2x0_1 7_2	1	$\theta\mathbf{7_{60}}$	2x0_1 7_7	1
$\theta\mathbf{7_1}$	3x0_1	1	$\theta\mathbf{7_{31}}$	0_1 3_1 7_2	1	$\theta\mathbf{7_{61}}$	2x0_1 7_7	1
$\theta\mathbf{7_2}$	3x0_1	1	$\theta\mathbf{7_{32}}$	0_1 5_2 7_2	1	$\theta\mathbf{7_{62}}$	2x0_1 7_7	1
$\theta\mathbf{7_3}$	3x0_1	1	$\theta\mathbf{7_{33}}$	2x0_1 7_3	2	$\theta\mathbf{7_{63}}$	2x0_1 7_7	1
$\theta\mathbf{7_4}$	3x0_1	1	$\theta\mathbf{7_{34}}$	2x0_1 7_3	2	$\theta\mathbf{7_{64}}$	0_1 3_1 7_7	2
$\theta\mathbf{7_5}$	2x0_1 3_1	2	$\theta\mathbf{7_{35}}$	0_1 3_1 7_3	2	$\theta\mathbf{7_{65}}$	0_1 4_1 7_7	1

Table S1. Theta-curves, their constituent knots, and their unknotting numbers.

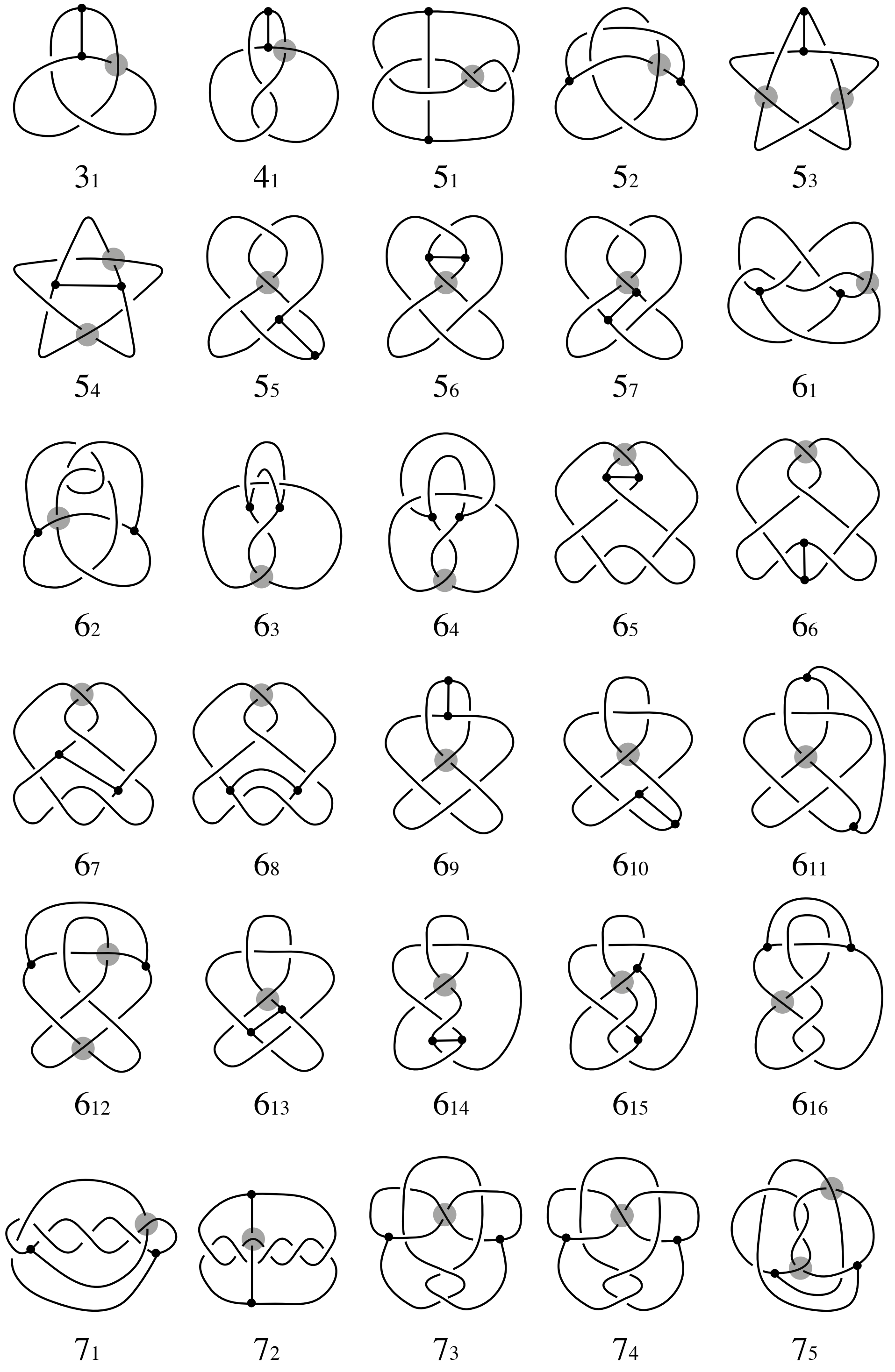

FIGURE S1. Theta-curves with their unknotting crossing changes shown in gray.

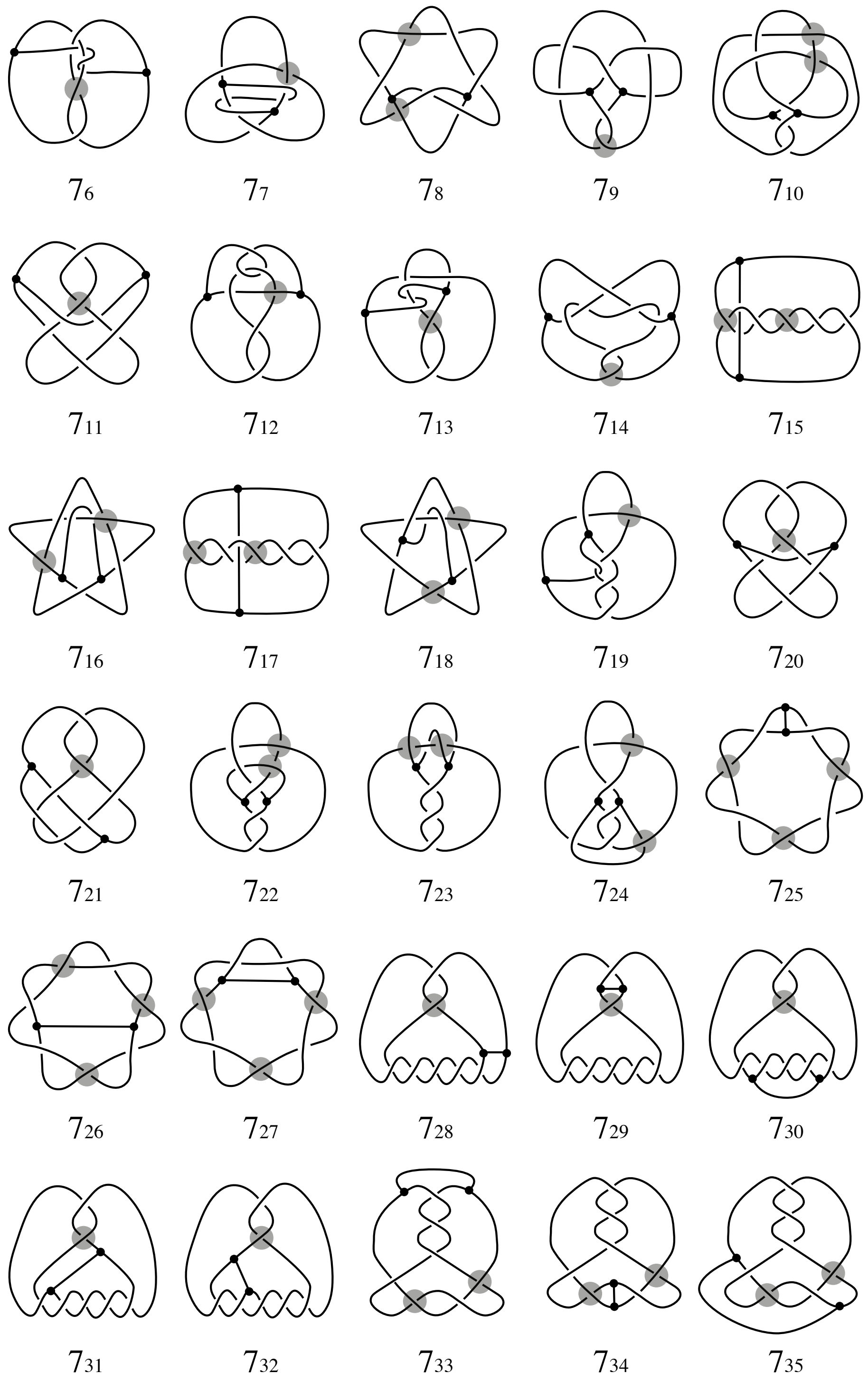

Figure S1. continued. θ-curves with their unknotting crossing changes shown in gray.

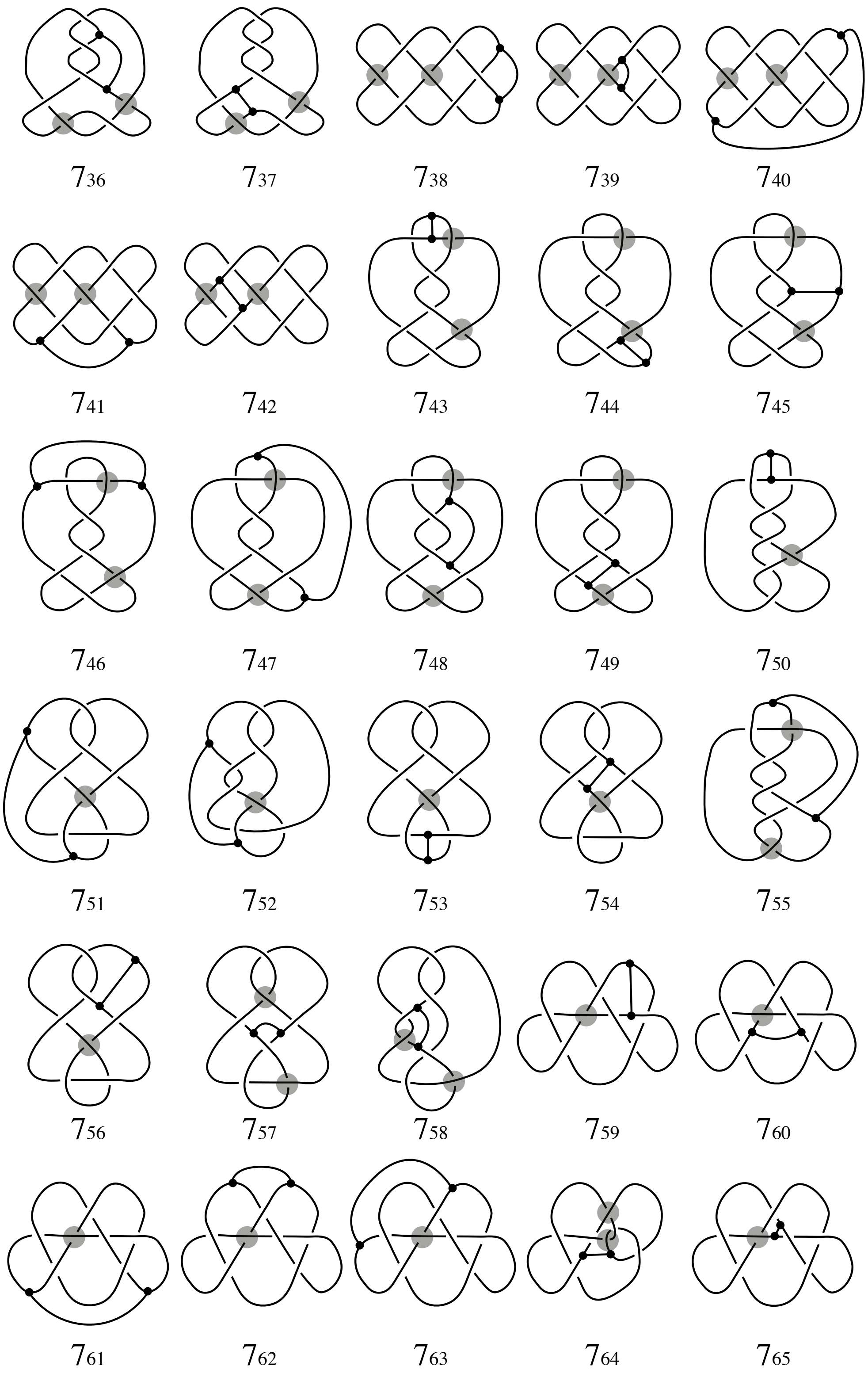

FIGURE S1. CONTINUED. θ-curves with their unknotting crossing changes shown in gray.
